# Thyroid function tests in patients taking thyroid medication in Germany: Results from the population-based Study of Health in Pomerania (SHIP)

**DOI:** 10.1186/1756-0500-3-227

**Published:** 2010-08-16

**Authors:** Anke Hannemann, Nele Friedrich, Robin Haring, Alexander Krebs, Henry Völzke, Dietrich Alte, Matthias Nauck, Thomas Kohlmann, Hans-Christof Schober, Wolfgang Hoffmann, Henri Wallaschofski

**Affiliations:** 1 Institute of Clinical Chemistry and Laboratory Medicine, Ernst-Moritz-Arndt University Greifswald, Ferdinand-Sauerbruch-Straße, 17475 Greifswald, Germany; 2 Institute of Community Medicine, Ernst-Moritz-Arndt University Greifswald, Walther-Rathenau-Straße 48, 17487 Greifswald, Germany; 3 Department of Internal Medicine I, Klinikum Südstadt Rostock, Südring 81, 18059 Rostock, Germany

## Abstract

**Background:**

Studies from iodine-sufficient areas have shown that a high proportion of patients taking medication for thyroid diseases have thyroid stimulating hormone (TSH) levels outside the reference range. Next to patient compliance, inadequate dosing adjustment resulting in under- and over-treatment of thyroid disease is a major cause of poor therapy outcomes. Using thyroid function tests, we aim to measure the proportions of subjects, who are under- or over-treated with thyroid medication in a previously iodine-deficient area.

**Findings:**

Data from 266 subjects participating in the population-based Study of Health in Pomerania (SHIP) were analysed. All subjects were taking thyroid medication. Serum TSH levels were measured using immunochemiluminescent procedures. TSH levels of < 0.27 or > 2.15 mIU/L in subjects younger than 50 years and < 0.19 or > 2.09 mIU/L in subjects 50 years and older, were defined as decreased or elevated, according to the established reference range for the specific study area. Our analysis revealed that 56 of 190 (29.5%) subjects treated with thyroxine had TSH levels outside the reference range (10.0% elevated, 19.5% decreased). Of the 31 subjects taking antithyroid drugs, 12 (38.7%) had TSH levels outside the reference range (9.7% elevated, 29.0% decreased). These proportions were lower in the 45 subjects receiving iodine supplementation (2.2% elevated, 8.9% decreased). Among the 3,974 SHIP participants not taking thyroid medication, TSH levels outside the reference range (2.8% elevated, 5.9% decreased) were less frequent.

**Conclusion:**

In concordance with previous studies in iodine-sufficient areas, our results indicate that a considerable number of patients taking thyroid medication are either under- or over-treated. Improved monitoring of these patients' TSH levels, compared to the local reference range, is recommended.

## Background

Hypothyroidism and hyperthyroidism are common diseases, which are treated with hormone replacement or antithyroid drugs, respectively. Applied therapies are targeted at adjusting the serum thyroid stimulating hormone (TSH) concentration to values within the reference range. However, studies from the U.S. [1,2] and the U.K. [3,4] investigating therapy outcome have found that a considerable proportion of patients taking medication for thyroid diseases have TSH levels outside the reference range. In the U.S. National Health and Nutrition Examination Study (NHANES), about one-third of the 820 participants who reported having thyroid disease or taking thyroid medication, had TSH levels outside the reference range [2]. In the Colorado Thyroid Disease Prevalence Study, 40% of the 1,525 subjects undergoing thyroid hormone replacement or antithyroid drug therapy had either elevated or decreased TSH levels [1]. Next to patient compliance, inadequate dosing adjustment resulting in under- and over-treatment of thyroid diseases is a major cause of poor therapy outcomes. In two studies conducted in general medical practices in the U.K. [3,4], more than 20% of patients taking thyroxine had suppressed TSH levels. The authors concluded that taking medication for hypothyroidism is, therefore, associated with an elevated risk for hyperthyroidism [3,4].

Previously, Germany was an iodine-deficient region [5]. While salt iodinization was introduced in the U.S. in 1924 [6], Germany's first prophylactic measures were not introduced until the 1980s [5]. These measures were mandatory in the former eastern part and voluntary in the former western part of the country. After reunification in 1989, the principle of voluntary iodine prophylaxis was adopted throughout the entire country. At the beginning of the 1990s, Germany's iodine supply was still inadequate, causing a high prevalence of diseases related to iodine deficiency [5]. Since the use of iodised salt became more widespread in 1994, iodine intake has improved markedly and in 1996, Germany's iodine supply was almost adequate [5]. Today, with the exception pregnant women and other subgroups with higher demand [7], an adequate iodine intake is assured in the German population.

Among the 4,310 participants of the Study of Health in Pomerania (SHIP), 35.9% were diagnosed with goitre, and 20.2% with thyroid nodules [8]. The prevalence of hypo- and hyperthyroidism among subjects without known thyroid disease was 1.2%, and 2.2%, respectively. The high prevalence of thyroid disorders can be explained by the insufficient iodine supply in previous decades [8]. Under- or over-treatment due to inadequate thyroid therapy monitoring may result in a disturbance of the thyroid hormone profile. Therefore, we aim to examine thyroid function in subjects taking thyroid medication in West Pomerania, a previously iodine-deficient area of Europe.

## Methods

### Study Population

SHIP is a population-based study, conducted in West Pomerania, in northeast Germany. At the time of sampling (1996), the entire population living in the area was 158,864 adults between 20-79 years. Using a two-stage cluster sampling method, adopted from the WHO MONICA Project Augsburg, Germany, a sample of 7,008 men and women was selected, of which 4,310 individuals participated (68.8% of eligible subjects) [9]. All participants gave written informed consent. Data collection started in October 1997 and was finished in May 2001. The study conformed to the principles of the Declaration of Helsinki, as reflected by an *a priori *approval of the Ethics Committee of the Board of Physicians Mecklenburg-Pomerania at the University of Greifswald.

Information on medical history, behavioural and socio-demographic characteristics was obtained using a computer-aided personal interview. Of 4,310 SHIP participants, 285 reported thyroid medication use according to the anatomical therapeutic chemical (ATC) code for thyroid therapy (H03). None of these 285 participants were affected by thyroid cancer, central hypothyroidism, or pituitary disease. Eight subjects reported a history of radio-iodine therapy, 86 reported a history of thyroid surgery, and two reported a history of both. Among the subjects for whom the year of surgery is known (n = 83), almost all (96.4%) underwent thyroid surgery more than one year prior to the SHIP examination. Further details on the start date, duration, and medical indication for thyroid therapy were not collected.

We excluded nine subjects from the analysis. Two subjects could not be assigned to any thyroid medication category (thyroxine, antithyroid drugs or iodine supplementation), and seven were excluded for using combinations of these medications. Another ten of the remaining 276 subjects were excluded because their TSH level had not been measured. This resulted in a study population of 266 subjects taking thyroid medication with TSH measurements. Among those, 219 were females between the ages of 22 and 80 years (mean 55.1 years), and 47 were males between the ages of 28 and 81 years (mean 60.0 years). For the purpose of comparison, we contrasted their results with those of the participants not taking thyroid medication, including subjects with unknown thyroid disease. Among those participants, TSH levels were available for 3,974 of 4,025 subjects. That population included 1,932 females between the ages of 20 and 81 years (mean 47.9 years) and 2,042 males between the ages 20 and 80 years (mean 50.6 years).

### Laboratory methods

Between 7:00 a.m. and 4:00 p.m., non-fasting blood samples were drawn from the cubital vein of subjects in the supine position. Thyroid function was evaluated by measurement of serum TSH, free triiodothyronine (FT_3_), free thyroxine (FT_4_), and auto-antibodies to thyroperoxidase (TPOAb). We focused on the TSH level in our analysis, as it provides adequate information for screening and monitoring of thyroxine replacement or suppression therapy [10].

Serum TSH levels were measured using an immunochemiluminescent assay (Byk Sangtec Diagnostica GmbH, Frankfurt, Germany). The assay was performed according to the manufacturer's recommendations on a LIA-mat analyzer. The functional sensitivity of the assay is specified as 0.02 mIU/L. The reference range provided by the manufacturer was 0.3-3.0 mIU/L, and the reference range specifically established for the SHIP population was 0.21-2.15 mIU/L in subjects younger than 50 years and 0.19-2.09 mIU/L in subjects 50 years and older [11]. The SHIP-specific reference interval represents the central 95% range between the 2.5^th ^and 97.5^th ^percentile from 1,488 subjects without known thyroid disease and without a diagnosed thyroid disorder as assessed by the SHIP examination [11]. Values outside the reference intervals were considered decreased or elevated. A TSH level less than 0.1 mIU/L was considered suppressed.

### Statistical Methods

Due to the skewed distribution of the TSH values, we routinely report median values. Median, 25^th^, 75^th ^percentiles and the range of TSH levels are presented by sex and thyroid medication type. Additionally, the proportion of subjects with TSH levels outside the reference interval is expressed as percent values. All statistical analyses were performed with SAS 9.1 (SAS Institute Inc., Cary, NC, USA).

## Results

**Figure 1:**
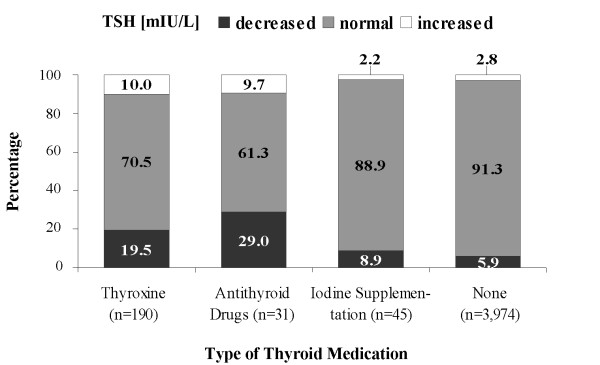
**Proportion of decreased, normal and increased serum thyroid stimulating hormone (TSH) levels according to the local reference range ( < 50 years: 0.27-2.15 mIU/L; ≥ 50 years: 0.19-2.19 mIU/L) by type of thyroid medication**.

The majority (71%) of the 266 SHIP participants taking thyroid medication were treated with thyroxine (Table 1). Another 12% of subjects took antithyroid drugs, and 17% received iodine supplementation (Table 1 and Figure 1). Participants were not asked for their daily dosage of their thyroid medication, but they were asked for the name of the preparation. Thyroxine preparations taken by SHIP participants contained 25-200 μg levothyroxine (23.2% in combination with iodine, 8.9% in combination with liothyronine), antithyroid drugs contained 5-20 mg thiamazol and iodine preparations contained 100-200 μg iodine.

**Table 1:** TSH distribution in the study population separated by type of thyroid medication

Type of thyroid medication	TSH [mIU/L]				
	
	Min	25%	Median	75%	Max
**Thyroxine**					
Male (n = 36)	0.02	0.31	0.60	1.47	2.99
Female (n = 154)	0.02	0.25	0.47	1.10	11.30

**Antithyroid drugs**					
Male (n = 4)	0.02	0.07	0.54	5.34	2.99
Female (n = 27)	0.02	0.13	0.56	1.21	11.30

**Iodine supplementation**					
Male (n = 7)	0.27	0.35	0.65	0.99	1.76
Female (n = 38)	0.04	0.35	0.53	0.80	2.11

**None**					
Male (n = 2042)	0.02	0.43	0.65	0.94	54.00
Female (n = 1932)	0.02	0.46	0.69	1.01	86.00

TSH, serum thyroid stimulating hormone; Min, minimum; Max, maximum

In subjects taking thyroid medication, TSH levels ranged from 0.02 to 11.30 mIU/L. TSH levels were within the SHIP reference range in 70.5% of the subjects taking thyroxine, in 61.3% of those taking antithyroid drugs and in 88.9% of patients receiving iodine supplementation (Figure 1). TSH levels were elevated in 10.0% of subjects taking thyroxine, in 9.7% of those taking antithyroid drugs and in 2.2% of patients receiving iodine supplementation. Decreased TSH values were observed in 19.5% of subjects taking thyroxine, in 29.0% of those taking antithyroid drugs and in 8.9% of subjects receiving iodine supplementation. Suppressed TSH levels were found in 34 individuals of whom 71% were treated with thyroxine, followed by 26% taking antithyroid drugs, and 3% with iodine supplementation (data not shown).

**Figure 2:**
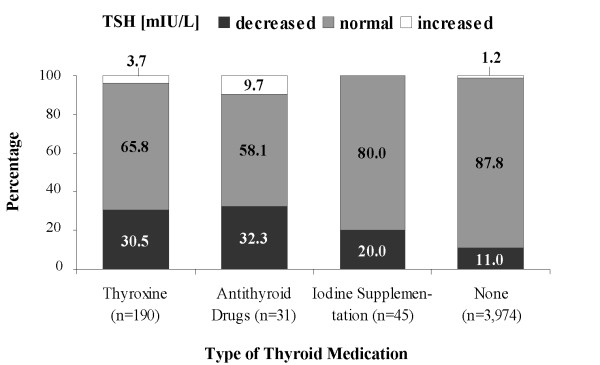
**Proportion of decreased, normal and increased serum thyroid stimulating hormone (TSH) levels according to the manufacturer's reference range ( all ages: 0.3-3.0 mIU/L) by type of thyroid medication**.

When the manufacturer's reference range was used instead of the SHIP-specific reference range, normal TSH levels were less frequent and decreased TSH levels were more frequent (Figure 2). This was observed for all classes of thyroid medication, while higher proportions of elevated TSH were only observed in patients using thyroxine or iodine supplementation. In subjects taking antithyroid drugs, the proportion of patients with elevated TSH levels was the same regardless of whether the SHIP-specific or the manufacturer's reference range was used.

In total, 27.4% of SHIP participants taking thyroid medication had TSH values outside the local reference range (8.6% elevated, 18.8% decreased). Using the manufacturer's reference range, 32.7% of the subjects had an elevated (3.8%) or a decreased (28.9%) TSH level. Among the SHIP participants not taking thyroid medication, the proportion of subjects with TSH levels outside the reference range was substantially lower (8.7% SHIP-specific, 12.2% manufacturer's).

## Discussion

As seen in previous studies [1,2,12], our results indicate that a considerable proportion of subjects taking thyroid medication are either under- or overtreated. About 27.4% of the SHIP participants taking thyroid medication had TSH levels outside the local reference range. Among the participants on thyroxine, nearly one-fifth (19.5%) were over-treated, as indicated by decreased TSH levels. Another 10.0% had elevated TSH values. In this group it was not possible to differentiate between poor compliance and under-treatment. In subjects taking antithyroid drugs, TSH levels can remain suppressed for weeks or months after the initiation of therapy [13]. Therefore, it would have been preferable to include the duration of treatment in the analysis. However, this information was not gathered, and such an analysis was not possible.

Iodine intake is the main determinant of regional patterns and prevalence of thyroid disorders [14]. In previously iodine-deficient regions, such as Germany, hyperthyroidism caused by goitre and thyroid nodules is common [8,15]. In regions with high or previously high iodine intake, such as the U.S. [16] or Iceland [14], a lower prevalence of hyperthyroidism and a higher prevalence of hypothyroidism have been reported [2,14]. Differences in iodine intake and thyroid disorders are reflected in the reference intervals for TSH. The SHIP-specific reference interval is lower than those for the general U.S. population and the manufacturer's [11]. It seems that SHIP patients taking thyroxine are adjusted to the local reference range, with less subjects classified as over-treated than when using the manufacturer's reference range (19.5% SHIP-specific vs. 30.5% manufacturer's).

Despite regional disparities in the prevalence and patterns of thyroid diseases, we found comparable proportions of subjects with elevated or decreased TSH levels between the SHIP region, the U.S. and the UK. About 27.4% of subjects from our study population had elevated or decreased TSH levels, compared to 33% in the U.S. NHANES study [2]. Among the SHIP participants treated with thyroxine, about one-fifth (19.5%) were over-treated, which is equal to the proportions observed in the U.K (21% [3] and 23% [4]).

In Germany, huge efforts have been made to raise awareness and increase and stabilise the iodine intake in the population. Those efforts have improved the situation markedly. However, efforts should also be made to improve thyroid therapy monitoring. As previously shown, inadequate or excessive thyroxine dosing causes mild thyroid hypo- and hyperfunction [3].

The standard treatment in patients with hypothyroidism is thyroxine. However, in the absence of screening programs, patients with thyroid failure may remain undiagnosed [12,17]. Even mild thyroid hypofunction may have adverse effects on circulating lipid levels and may increase the risk of ischemic heart disease [2,18,19]. An elevated TSH level, characteristic of hypothyroidism, was seen in 10.0% of the SHIP participants taking thyroxine. A decreased TSH level, which represents a risk factor for developing atrial fibrillation [20] and osteoporosis [21], was observed in 19.5% of SHIP participants taking thyroxine. By exact dose monitoring, adverse effects resulting from under- or over-treatment can be minimised. Furthermore, there may be a positive impact on the cost of thyroid medications, which accounted for 296.5 million € in Germany in 2007 [22].

Potential limitations of our study arise from the use of medications for non-thyroid diseases in our study population. Circulating concentrations of thyroid hormones are altered by several drugs, such as lithium, amiodarone, testosterone, and oestrogen [12,15,23]. Lithium and amiodarone are both associated with increases in TSH levels [12]. However, these two substances were rarely used by SHIP participants. Among the 266 study subjects taking thyroid medication, lithium was used by two, and amiodarone was not used by any subject. Therefore, the impact of these drugs on the population level is assumed to be much smaller than that of the effects of under- and over-treatment. The same reasoning applies to the use of testosterone, because only 1 of 47 men taking thyroid medication was also using testosterone. Oestrogen, in contrast, was widely used, with 51 of 219 women reporting concomitant use of thyroid medication and oestrogen. Since the oestrogen-TSH association has been shown to be weak [12], we do not suspect a relevant influence of this medication on our results. The timing of blood sampling is also unlikely to be a relevant influence on our results, as the pulsatile and circadian secretion of TSH is of minor importance in thyroid disease diagnostics [24]. Furthermore, we do not consider the missing data on indication for thyroid medication use or prescribed dose as a limitation, as the adverse effects of under- or over-treatment with thyroid medication are independent of these treatment characteristics.

Major limitations include the small study population and the low proportion of male subjects, which are due to the population-based study design. Moreover, information on the duration of thyroid medication intake was not collected. We know that among participants who reported a history of thyroid surgery, less than 5% underwent an operation in the year prior to the SHIP examination. Among the remaining subjects, though, we cannot rule out that a significant number started thyroid therapy shortly before participating in SHIP. If this were the case, those subjects were probably still undergoing dose adjustment at the time of the SHIP examination and may not have reached a stable TSH level, which may have introduced some bias in our study.

## Conclusions

We suggest that general practitioners and other care providers for patients taking thyroid medication should more carefully monitor the effects of therapy, adjust dosages and encourage compliance. Further research, including a health economics evaluation, is needed to optimise thyroid disease monitoring.

## Competing interests

The authors declare that they have no competing interests.

## Authors' contributions

HW, DA, NF and RH contributed ideas for the data analysis. AH, NF and DA performed and supervised the data analysis. MN and AK performed and supervised the laboratory measurements. HW, HV, MN, TK, WH and HCS contributed to the interpretation of the results and the discussion. AH drafted the manuscript and wrote the final version together with all other co-authors. All authors read and approved the final manuscript.

## References

[B1] Canaris GJ Manowitz NR Mayor G Ridgway EC The Colorado thyroid disease prevalence study Arch Intern Med 2000 160 526 534 10.1001/archinte.160.4.526 10695693

[B2] Hollowell JG Staehling NW Flanders WD Hannon WH Gunter EW Spencer CA Braverman LE Serum TSH, T(4), and thyroid antibodies in the United States population (1988 to 1994): National Health and Nutrition Examination Survey (NHANES III) J Clin Endocrinol Metab 2002 87 489 499 10.1210/jc.87.2.489 11836274

[B3] Parle JV Franklyn JA Cross KW Jones SR Sheppard MC Thyroxine prescription in the community: serum thyroid stimulating hormone level assays as an indicator of undertreatment or overtreatment Br J Gen Pract 1993 43 107 109 8323787 PMC1372330

[B4] De Whalley P Do abnormal thyroid stimulating hormone level values result in treatment changes? A study of patients on thyroxine in one general practice Br J Gen Pract 1995 45 93 95 7702890 PMC1239143

[B5] Meng W Schindler A Delange F, Robertson A, McLoughney E, Gerasimov G Iodine Supply in Germany Elimination of Iodine Deficiency Disorders (IDD) in Central and Eastern Europe, the Commonwealth of Independent States and the Baltic States. Proceedings of a Conference held in Munich, Germany: 3-6 September 1997; Munich 1998 World Health Organization 21 30

[B6] WHO/UNICEF/ICCIDD Progress Towards the Elimination of Iodine Deficiency Orders (IDD), Geneva 1999

[B7] Meng W Scriba PC Jodversorgung in Deutschland. Probleme und erforderliche Maßnahmen: Update 2002 Deutsches Ärzteblatt 2002 99 2560 2564

[B8] Volzke H Ludemann J Robinson DM Spieker KW Schwahn C Kramer A John U Meng W The prevalence of undiagnosed thyroid disorders in a previously iodine-deficient area Thyroid 2003 13 803 810 10.1089/105072503768499680 14558922

[B9] John U Greiner B Hensel E Ludemann J Piek M Sauer S Adam C Born G Alte D Greiser E Haertel U Hense HW Haerting J Willich S Kessler C Study of Health In Pomerania (SHIP): a health examination survey in an east German region: objectives and design Soz Praventivmed 2001 46 186 194 10.1007/BF01324255 11565448

[B10] Ross DS Daniels GH Gouveia D The use and limitations of a chemiluminescent thyrotropin assay as a single thyroid function test in an out-patient endocrine clinic J Clin Endocrinol Metab 1990 71 764 769 10.1210/jcem-71-3-764 2394778

[B11] Volzke H Alte D Kohlmann T Ludemann J Nauck M John U Meng W Reference intervals of serum thyroid function tests in a previously iodine-deficient area Thyroid 2005 15 279 285 10.1089/thy.2005.15.279 15785248

[B12] Aoki Y Belin RM Clickner R Jeffries R Phillips L Mahaffey KR Serum TSH and total T4 in the United States population and their association with participant characteristics: National Health and Nutrition Examination Survey (NHANES 1999-2002) Thyroid 2007 17 1211 1223 10.1089/thy.2006.0235 18177256

[B13] Todd CH Management of thyroid disorders in primary care: challenges and controversies Postgrad Med J 2008 85 655 659 10.1136/pgmj.2008.077701 20075403

[B14] Laurberg P Pedersen KM Hreidarsson A Sigfusson N Iversen E Knudsen PR Iodine intake and the pattern of thyroid disorders: a comparative epidemiological study of thyroid abnormalities in the elderly in Iceland and in Jutland, Denmark J Clin Endocrinol Metab 1998 83 765 769 10.1210/jc.83.3.765 9506723

[B15] Reiners C Wegscheider K Schicha H Theissen P Vaupel R Wrbitzky R Schumm-Draeger PM Prevalence of thyroid disorders in the working population of Germany: ultrasonography screening in 96,278 unselected employees Thyroid 2004 14 926 932 10.1089/thy.2004.14.926 15671771

[B16] Hollowell JG Staehling NW Hannon WH Flanders DW Gunter EW Maberly GF Braverman LE Pino S Miller DT Garbe PL DeLozier DM Jackson RJ Iodine nutrition in the United States. Trends and public health implications: iodine excretion data from National Health and Nutrition Examination Surveys I and III (1971-1974 and 1988-1994) J Clin Endocrinol Metab 1998 83 3401 3408 10.1210/jc.83.10.3401 9768638

[B17] Parle JV Franklyn JA Cross KW Jones SC Sheppard MC Prevalence and follow-up of abnormal thyrotrophin (TSH) concentrations in the elderly in the United Kingdom Clin Endocrinol (Oxf) 1991 34 77 83 10.1111/j.1365-2265.1991.tb01739.x 2004476

[B18] Hak AE Pols HA Visser TJ Drexhage HA Hofman A Witteman JC Subclinical hypothyroidism is an independent risk factor for atherosclerosis and myocardial infarction in elderly women: the Rotterdam Study Ann Intern Med 2000 132 270 278 10681281 10.7326/0003-4819-132-4-200002150-00004

[B19] Morris MS Bostom AG Jacques PF Selhub J Rosenberg IH Hyperhomocysteinemia and hypercholesterolemia associated with hypothyroidism in the third US National Health and Nutrition Examination Survey Atherosclerosis 2001 155 195 200 10.1016/S0021-9150(00)00537-2 11223442

[B20] Sawin CT Geller A Wolf PA Belanger AJ Baker E Bacharach P Wilson PW Benjamin EJ D'Agostino RB Low serum thyrotropin concentrations as a risk factor for atrial fibrillation in older persons N Engl J Med 1994 331 1249 1252 10.1056/NEJM199411103311901 7935681

[B21] Stall GM Harris S Sokoll LJ Dawson-Hughes B Accelerated bone loss in hypothyroid patients overtreated with L-thyroxine Ann Intern Med 1990 113 265 269 2375563 10.7326/0003-4819-113-4-265

[B22] Ziegler R Schwalbe U Schwabe U, Paffrath D Schilddrüsentherapeutika Arzneiverordnungs-Report 2008 2008 Heidelberg: Springer Medizin Verlag 833 842

[B23] Meikle AW The interrelationships between thyroid dysfunction and hypogonadism in men and boys Thyroid 2004 14 Suppl 1 17 25 10.1089/105072504323024552 15142373

[B24] Brabant G Pulsatile and circadian TSH secretion. Clinical relevance? Internist (Berl) 1998 39 619 622 10.1007/s001080050223 9677521

